# Clinical profile and problems of management of 108 cases of germ cell tumours of testis at Institute Rotary Cancer Hospital, All India Institute of Medical Sciences New Delhi 1985-1990.

**DOI:** 10.1038/bjc.1993.105

**Published:** 1993-03

**Authors:** V. Raina, N. K. Shukla, G. K. Rath, N. P. Gupta, M. C. Mishra, T. K. Chaterjee, A. K. Kripalani

**Affiliations:** Institute Rotary Cancer Hospital, All India Institute of Medical Sciences, Ansari Nagar, New Delhi.

## Abstract

A retrospective analysis of 108 cases of primary germ cell tumours of testis seen over a 6 year period at Institute Rotary Cancer Hospital of All India Institute of Medical Sciences, New Delhi is presented. There were 45 (42%) cases of seminoma and 63 (48%) of non-seminomatous germ cell tumours (NSGCT). The median age at presentation was 35 and 30 years respectively. Almost half (56) patients presented in advanced stage (stages IIc-IV). Tumours in undescended testis formed an important subgroup (14%). The standard approach of treatment was radiotherapy in stages I & II seminomas and chemotherapy in bulky seminomas and metastatic NSGCT. Chemotherapy protocols used were VAB-6 and PVB. Although a policy of surveillance has been practised for stage I NSGCT, it is debatable whether it is universally suitable for our patients. The results of treatment in low volume disease are comparable to that in the west but the management of bulky disease requires a more aggressive approach. Unfortunately only 74 out of 108 (68.5%) patients were able to complete the treatment prescribed. Most of the defaulters were from the chemotherapy group because of inability to afford the drugs. The probability of survival of those who completed treatment was 0.77 at 4 years. Since testicular tumours are largely curable, a more vigorous policy of detection, follow up and treatment needs to be pursued. Better screening of children with undescended testis will reduce cancer in this group. Failure to provide chemotherapy to all patients is particularly unfortunate for a curable disease like testis cancer.


*Br. J. Cancer* (1993), **67**, 573–577 © Macmillan Press Ltd., 1993

### Clinical profile and problems of management of 108 cases of germ cell tumours of testis at Institute Rotary Cancer Hospital, All India Institute of Medical Sciences New Delhi 1985–1990

V. Raina, N.K. Shukla, G.K. Rath, N.P. Gupta, M.C. Mishra, T.K. Chaterjee & A.K. Kripalani

*Institute Rotary Cancer Hospital, All India Institute of Medical Sciences, Ansari Nagar, New Delhi 110 029, India.*

**Summary** A retrospective analysis of 108 cases of primary germ cell tumours of testis seen over a 6 year period at Institute Rotary Cancer Hospital of All India Institute of Medical Sciences, New Delhi is presented. There were 45 (42%) cases of seminoma and 63 (48%) of non-seminomatous germ cell tumours (NSGCT). The median age at presentation was 35 and 30 years respectively. Almost half (56) patients presented in advanced stage (stages IIc–IV). Tumours in undescended testis formed an important subgroup (14%). The standard approach of treatment was radiotherapy in stages I & II seminomas and chemotherapy in bulky seminomas and metastatic NSGCT. Chemotherapy protocols used were VAB-6 and PVB. Although a policy of surveillance has been practised for stage I NSGCT, it is debatable whether it is universally suitable for our patients. The results of treatment in low volume disease are comparable to that in the west but the management of bulky disease requires a more aggressive approach. Unfortunately only 74 out of 108 (68.5%) patients were able to complete the treatment prescribed. Most of the defaulters were from the chemotherapy group because of inability to afford the drugs. The probability of survival of those who completed treatment was 0.77 at 4 years. Since testicular tumours are largely curable, a more vigorous policy of detection, follow up and treatment needs to be pursued. Better screening of children with undescended testis will reduce cancer in this group. Failure to provide chemotherapy to all patients is particularly unfortunate for a curable disease like testis cancer.

Testicular tumours account for only about 1% of all cancers but are the most common neoplasms in the 15–35 year old age group (Einhorn *et al.*, 1977). This cancer thus has the potential of resulting in the loss of productive years of life. With the advent of modern chemotherapeutic agents most importantly DDP, testicular cancers have become largely curable. Although the natural history of this neoplasm is well known in the west, its natural history in India which currently has a population of about 850 million is not well documented. The aim of this paper is to put on record the clinical features, treatment and problems of management of 108 cases of primary germ cell tumours of testis that were seen over a 6 year period at Institute Rotary Cancer Hospital (IRCH) of All India Institute of Medical Sciences (AIIMS) New Delhi. This is a regional cancer centre and gets referrals from north and north central India involving 50 million, a population almost equal to that of UK.

### Materials and methods

Case records of all patients with testicular tumours that were registered at IRCH between Jan 1985 and Dec 1990 were analysed. These patients had a proven histological diagnosis, a complete clinical examination and staging. One hundred and thirteen cases of primary testicular tumours were registered. Five of these patients had non germ cell tumours and have been excluded from analysis. Besides clinical examination, all patients had complete hemogram, routine biochemistry, markers like b-human chorionic gonadotrophin and alpha fetoprotein, radiograph of the chest, and CT scans. Patients were staged using the Royal Marsden Hospital classification (Peckham *et al.*, 1979). Histological staging was done using the WHO classification (Mostofi & Sobin, 1977). Probability of survival was calculated in those patients who had completed the planned treatment.

Correspondence: V. Raina, Additional Professor of Medical Oncology, Institute Rotary Cancer Hospital, All India Institute of Medical Sciences, New Delhi 110 029.
Received 20 November 1991; and in revised form 7 September 1992.

### *Seminomas*

There were 45 cases of seminoma. These presented at a median age of 35 years (range 19–74 years). There were proportionately more cases in early stages i.e. 28 out of 36 (stages I–IIb).

### *NSGCT*

There were 63 cases in this category. The commonest in this group was immature teratoma in 52 (82%) cases. The median age of these tumours was 30 years with a range of 3–50 years. NSGCT were distributed more or less evenly with respect to stages.

### *Undescended or partially descended testis*

There were 15 cases in this category accounting for 14% of all cases of primary germ cell tumours of testis. Ten patients had unilateral and five bilateral abnormalities of descent. All these patients had either abdominal swelling or pain as the presenting feature and had large abdominal masses or ascites. Extrapolating the Royal Marsden Hospital classification, all patients in this category were in either stage IIc or IId. The median age of these patients was 29 years with a range of 20–43 years. Six patients had seminoma and nine NSGCT.

### *Mode of presentation*

In 74 (71%) patients the duration of symptoms was more than six months. An analysis of the mode of presentation revealed testicular swelling to be the feature in 70 (65%) cases. The second commonest mode of presentation was abdominal swelling i.e. in 28 cases (25%). The other ten patients had supraclavicular node (five), pulmonary (three), neurological (one), skeletal (one) metastasis as the presenting features. The distribution of staging with reference to histological type is shown in Table I. It is obvious from the table that almost half (56) patients were in advanced stage, i.e. stages IIc–IV. All patients had CT scanning, hence understaging was unlikely. Exact staging was not known in seven cases because of incomplete information. Of the 27

**Table I** Stages and histology of testicular tumours at IRCH

| *Stage* | *Seminoma* | *NSGCT* | *Overall* |
|---|---|---|---|
| I | 20 | 12 | 32 |
| II | 8 | 14 | 22 |
| III | 2 | 3 | 5 |
| IV | 6 | 21 | 27 |
| Incomplete staging | 3 | 4 | 7 |
| Undescended | 6 | 9 | 15 |
| Total | 45 | 63 | 108 |
| Treatment completed | 35 | 39 | 74 (68.5%) |

patients with stage IV disease six had pulmonary, 13 hepatic, eight with both hepatic and pulmonary metastasis.

**Treatment strategies and results**

All 93 patients who had normally situated testis had orchidectomy. In 81 patients this was a high inguinal orchidectomy done either by the referring surgeon or at our hospital. In others scrotal orchidectomy was done in peripheral hospitals and patients were referred thereafter. Of the 15 patients with abnormalities of descent 14 had undergone laparotomy for purposes of histological diagnosis and treatment. In one patient the diagnosis was made on a trucut biopsy of the abdominal mass.

*Seminoma*

There were 19 cases of stage I seminoma. These were treated by conventional radiotherapy involving two anterior and posterior portals encompassing bilateral para-aortic and pelvic lymph nodes delivering a dose of 30 Gy in 4 weeks with cobalt 60 beam. The scrotum and inguinal lymph nodes were included in treatment portals in patients having extracapsular spread or in patients with a previous history of surgery in the inguinoscrotal region. Nine patients who came from distant regions were lost to follow up at variable periods after completing radiotherapy. Ten patients continue to have no evidence of disease at a median fiollow up of 18 months (range 12–76 months). There were 26 cases of stages II–IV seminoma (including five with abnormalities of descent and three with incomplete information on staging). Six patients who had non-bulky disease (IIA & IIB) were treated with radiotherapy and 20 patients with bulky or advanced disease started on primary chemotherapy, 12 on VAB-6 and eight on PVB protocols (Vugrin *et al.*, 1981 and Einhorn & Donohue, 1977). Ten patients did not complete their treatment (six radiotherapy and four chemotherapy) due to expenses involved and returned to their native places. Of the 16 patients who completed their prescribed chemotherapy 12 achieved complete remission and are disease free at a median of 18 months (range 12–60 months). Four patients died of progressive disease. The probability of survival in 35 patients who completed their treatment is shown in Figures 1 and 2.

**Figure 1** Probability of survival seminoma (stages II–IV) 16 pts.

**Figure 2** Probability of survival seminoma all stages (35 pts).

*NSGCT*

There were 12 cases of stage I NSGCT. Ten patients were put on surveillance. Six patients continue to be disease free at a median of 12 months (range 10–56 months). One patient relapsed at 9 months. This relapse was detected on periodic marker estimation. Patient was given three courses of BEP (Bleomycin, Etoposide and DDP) chemotherapy and promptly went into remission. Three patients have been lost to follow up. One patient was treated by pelvic radiotherapy and is alive and disease free at 30 months. Another underwent retroperitoneal lymph node dissection (RPLND), was found to be disease free and continues to be so at 36 months. There were 51 cases of advanced NSGCT (stages II–IV including nine with abnormalities of descent and four with incomplete information on staging). Only 27 of these patients completed their chemotherapy. Others either declined chemotherapy or dropped out after having one or two courses. Twenty-one (77%) of 27 patients went into complete remission. Of these 17 achieved complete remission that was achieved with chemotherapy alone. In four patients partial remission that was ahieved with chemotherapy was converted into complete remission by resection of residual tumour in the abdomen. The median follow up of these patients is 18 months with a range of 8–60 months. One patient died due to chemotherapy toxicity and two died of progressive disease. Three patients achieved only partial remission. They had significant residual disease at multiple sites, were not considered suitable for resection and died in spite of salvage chemotherapy. The probability of survival in those who completed treatment is shown in Figures 3 and 4.

Two different chemotherapy protocols were used. These were PVB (DDP, Vinblastine, Bleomycin) and VAB-6 (DDP, Vinblastine, Actinomycin-D, Bleomycin, Cyclophosphamide). A total of 62 patients of either seminoma or NSGCT were started on chemotherapy. Of these 17 patients did not complete chemotherapy and are not evaluable with regard to response. The major reason was the cost of chemotherapy. Although the state health services and IRCH are aimed to provide free treatment to all patients, it has actually not been possible to do so because of the cost of anti-cancer drugs, most of which are still imported. Patients who can afford are asked to buy their own drugs while as state funding or voluntary agency support is available for funding of the treatment of poor patients. This is not adequate for the most poor many of whom return home after becoming relatively symptom free after receiving one or two courses of chemotherapy. They do not respond or come back for treatment even after they are sent reminders. It is also frequently

**Figure 3** Probability of survival NSGCT (stages II–IV) 27 pts.

**Figure 4** Probability of survival NSGCT all stages (39 pts).

observed that the some of the poorest patients who are offered all chemotherapy free by voluntary agencies or from hospital are unable to complete their treatment either because they are not able to stay in Delhi due to pressing problems at home or do not understand the often difficult advice about chemotherapy and its toxicity because of almost nil education. Occasionally patients have no choice but to return home because all of them cannot be accomodated in the inpatient wards and are treated in day care instead. It is therefore unfortunate that only 74 out of 108 (68%) registered patients were able to complete their treatment. There were no deaths in stage I in either group. Some patients stopped coming for follow up even after completing their treatment. We therefore calculated the probability of survival. The data for the group as a whole is provided in Figure 5. Median survival was not attained in any group. The aim of showing the various probability of survival curves which have been estimated by Kaplan-Meier method is not to either compare the groups or to demonstrate the prognostic factors but to show the results in various groups of patients in the background of our working conditions. The number of patients at risk at various time periods have been provided in the figures and gives a fair idea of the loss of follow up.

### Discussion

As a result of remarkable advances in chemotherapy of testicular cancer it is obvious that this malignancy has become important to detect and treat. Testicular tumours though prevalant in the most productive years of the life are relatively uncommon. It is therefore difficult for one centre to have a large experience. Review of literature has revealed paucity of information on the pattern and management of testicular cancer in India. The report of 200 cases of testicular cancer from Post Graduate Institute of Medical Education and Research (PGIMER) Chandigarh, to our knowledge is the only major publication on this subject from India (Grover et al., 1985). Testicular tumours form about 1% of all tumours registered at IRCH. Population surveys indicate a crude incidence rate of 0.7 to 1 per 100,000 persons at Banglore, Bombay and Madras, the three centres from where information is available (Annual Report 1987, National Cancer Registry Programme, New Delhi). As population surveys from the rest of the country are not available the exact incidence of this disease is not known. Based on the rates of incidence from other major cities in India, it was expected that at least 350 new cases would be diagnosed every year. For the last 2 years i.e. 1990 and 1991 we have been registering about 60 cases every year which is slightly less than 20% of all possible new cases occurring in the population. It is obvious that all patients are not seen at our center. This could be either because they are not diagnosed or receive treatment in district hospitals or may be having self referrals to other major cancer centres in Bombay etc. It is also possible that many patients do not seek treatment. Notwithstanding all this, our is the largest referral centre for the population involved. It is interesting to note that almost three times as many (1000) new cases of germ cell tumour of testis are reported from UK every year, a population of 50 million, approximately the same as under discussion. Median age of our patients with seminoma is a decade lower than the reported western figures (Einhorn et al., 1977). The reasons for this are not clear but could be because older patients from distant places find it difficult to attend a tertiary hospital like ours and may be receiving treatment locally. The shorter life expectancy in India could also effect the age distribution of this condition. The lag period from symptoms to actual diagnosis is long i.e. more than 6 months in 70% cases. This is because of tendency to not to report symptoms involving a private organ and sparse availability of health care in certain areas. Quite often the only symptom is swelling, the condition is misdiagnosed as hydrocoele, and the patient reassured till testicular or abdominal pain or abdominal mass point to malignancy. This in turn results in patients being diagnosed in an advanced state. A look at our table indicates that only about a third (35) of patients were in stage I–IIb. This has an important bearing on the long term prognosis. Many studies have shown that high volume disease is the most important indicator of poor prognosis (Peckham et al., 1979). Tumours in undescended testis formed a proportion that is much higher than the current western figures and reflects the underdeveloped health services in peripheral areas (Einhorn et al., 1979). All patients who had tumours on undescended or maldescended testis had received either no or very poor primary education and belonged to very poor socio-economic class. Majority of such cases underwent laparotomy for diagnosis (14 out of 15). Resection in most of these cases was incomplete and patients required either chemotherapy or radiotherapy. This practice has changed over the last 2 years. Whenever this condition is suspected now (any abdominal mass with absent scrotal testes) tumour markers i.e. B-HCG and AFP are done, and histopathology obtained by tru-cut needle. If germ cell tumour is diagnosed, the patient is given appropriate chemotherapy and surgery reserved only for resistant residual masses or removal of intraabdominal testis.

With the availability of reliable markers and investigation facilities like whole body CT scanning and ultrasound the management of testicular tumours has become more rational

**Figure 5** Probability of survival all testicular cancer (74 pts).

in terms of early diagnosis, appropriate staging and better monitoring of patient's response to treatment. The number of patients receiving chemotherapy in each subgroup was small. Of the 74 patients who completed the planned treatment the probability of survival at 4 years was 0.77. This figure compares favourably with most western figures (Graham *et al.*, 1988). Since there have been no deaths in stage I in either group, the survival figures for advanced disease are likely to be worse. It is exactly these patients in whom a more aggressive treatment is required. It may appear that our response and survival results are not much inferior to western figures. We suggest that our figures be taken with caution because of high drop out rates of patients who did not complete their treatment. A majority of these patients had advanced or bulky disease.

Our main problems remain the (i) management of bulky disease, (ii) inability of patients to take complete treatment (iii) loss of follow up even in those who have become asymptomatic after initial treatment or have completed the prescribed treatment. This is particularly unfortunate for a potentially curable cancer like that of testis. The reasons are poverty, low education of patients and distances that they have to travel to because of the paucity of good cancer centres. The inability to take complete treatment is particularly great for patients with stage II bulky, III & IV. This is obviously because of the vast majority require chemotherapy which is expensive. Some of these patients return home without even informing us. We attempted to contact these patients by means of reply paid letters but the response was only 36%. This situation needs to be improved because testicular tumour patients are curable even in advanced stage. Our policy in stage I NSGCT is surveillance. This however may not be universally appropriate for our conditions, as surveillance presumes that the follow up be strict. Three of our patients on surveillance were lost to follow up. Also the cost of surveillance may be prohibitive in terms of frequent visits to hospital and need for regular CT scanning. CT scan and ultrasound facilities are available only in major hospitals in the cities. In such patients where it is feared that follow up may not be adequate, giving adjuvant chemotherapy or radiotherapy may be a good alternate option. This approach may be wholly appropriate for a certain section of our population and is currently being debated in our department. We do not currently favour retroperitoneal lymph node dissection for stage I NSGCT partly because of its significant morbidity in terms of failure of ejaculation which can become a major problem in young sexually active males and partly because it is difficult to convince asymptomatic patients to undergo major surgery. The current policy is to stratify patients into low, intermediate and high volume disease and to offer chemotherapy appropriate to the stage and volume as is practised by EORTC.

We thank Dr K.R. Sundaram PhD, Additional Professor of Biostatistics AIIMS for statistical advice, Mr Kailash of Computer Facility for computer assistance and Dr V. Raina who undertook the word processing himself.

### References

EINHORN, L. & DONOHUE, J.P. (1977). Cis-diamminedichloroplatinum, vinblastine and bleomycin combination chemotherapy in disseminated testicular cancer. *Ann. Intern. Med.*, **87**, 293-298.

EINHORN, L.H., DONOHUE, J.P., PECKHAM, M.J., WILLIAMS, S.D. & LOEHRER, P.J. (1977). Cancer of the testes. In DeVita V.T., Hellman S., & Rosenberg S.A. (eds). *Cancer: Principles and Practice of Oncology*. 979-1011. Lippincott.

GRAHAM, J., HARDING, M., MILL, L., KERR, D.J., RANKIN, E., CALMAN, K.C. & KAYE, S.B. (1988). Results of treatment of nonseminomatous germ cell tumours; 122 consecutive cases in the West of Scotland, 1981-1985. *Br. J. Cancer*, **57**, 182-185.

GROVER, R.K., KAUSHAL, V. & GUPTA, B.D. (1985). Testicular germ cell tumors. A review of 10 years' experience. *Cancer*, **56**, 1251-1256.

MOSTOFI, F.K., SOBIN, L.H. (1977). Histological Typing of Testis Tumors. *International Histological Classification of Tumors*, 16, Geneva: World Health Organisation.

NATIONAL CANCER REGISTRY PROGRAMME, *Annual Report 1987*. Indian Council of Medical Research. New Delhi.

PECKHAM, M.J., MCELWAIN, T.J., BARRET, A. & HENDRY, W.F. (1979). Combined management of malignant teratoma of the testis. *Lancet*, **ii**, 267-270.

VUGRIN, D., HERR, H.W., WHITMORE, W.F., SOGANI, P.C. & GOLBEY, R.B. (1981). VAB-6 combination chemotherapy in disseminated cancer of the testis. *Ann. Intern. Med.*, **95**, 59-61.


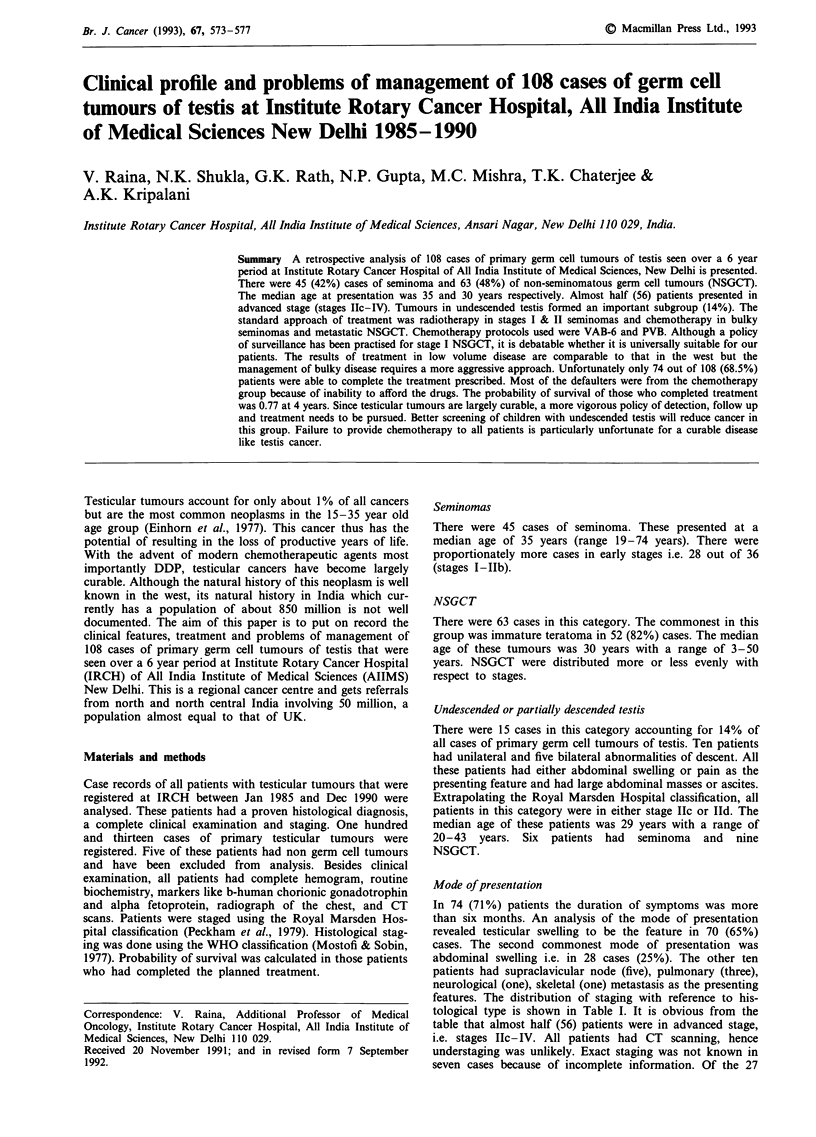

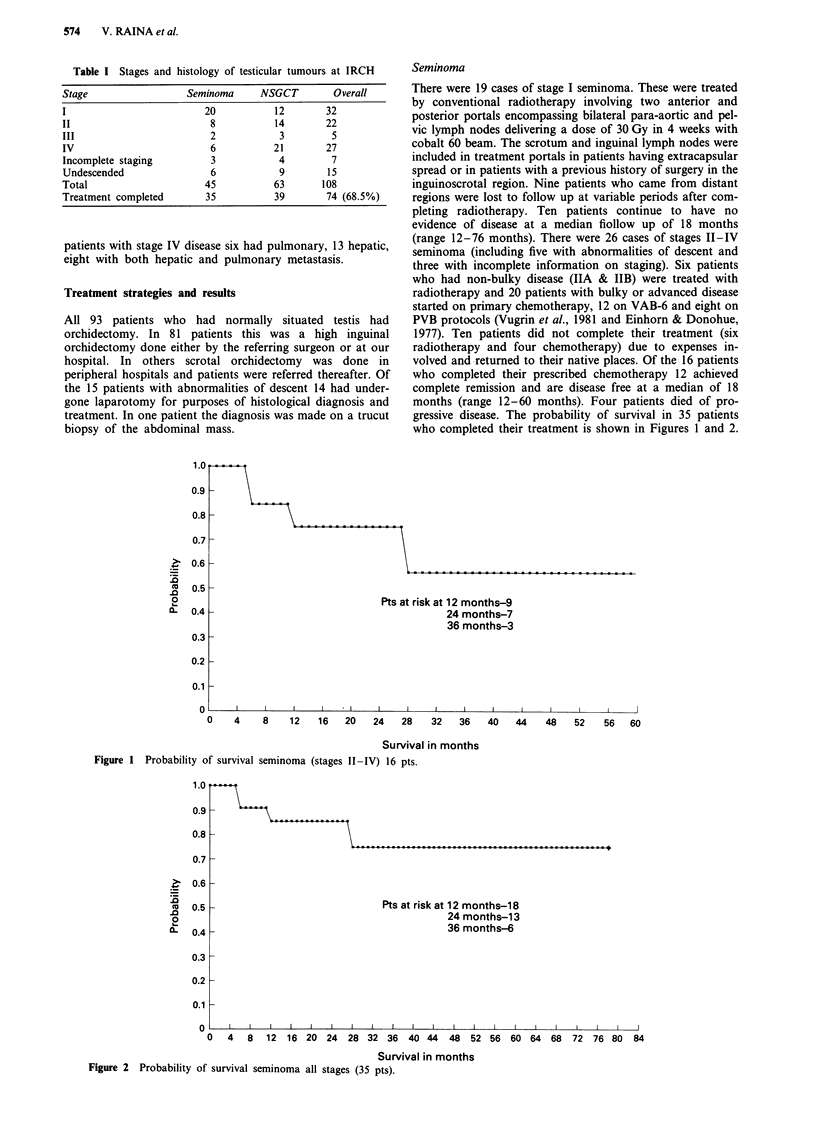

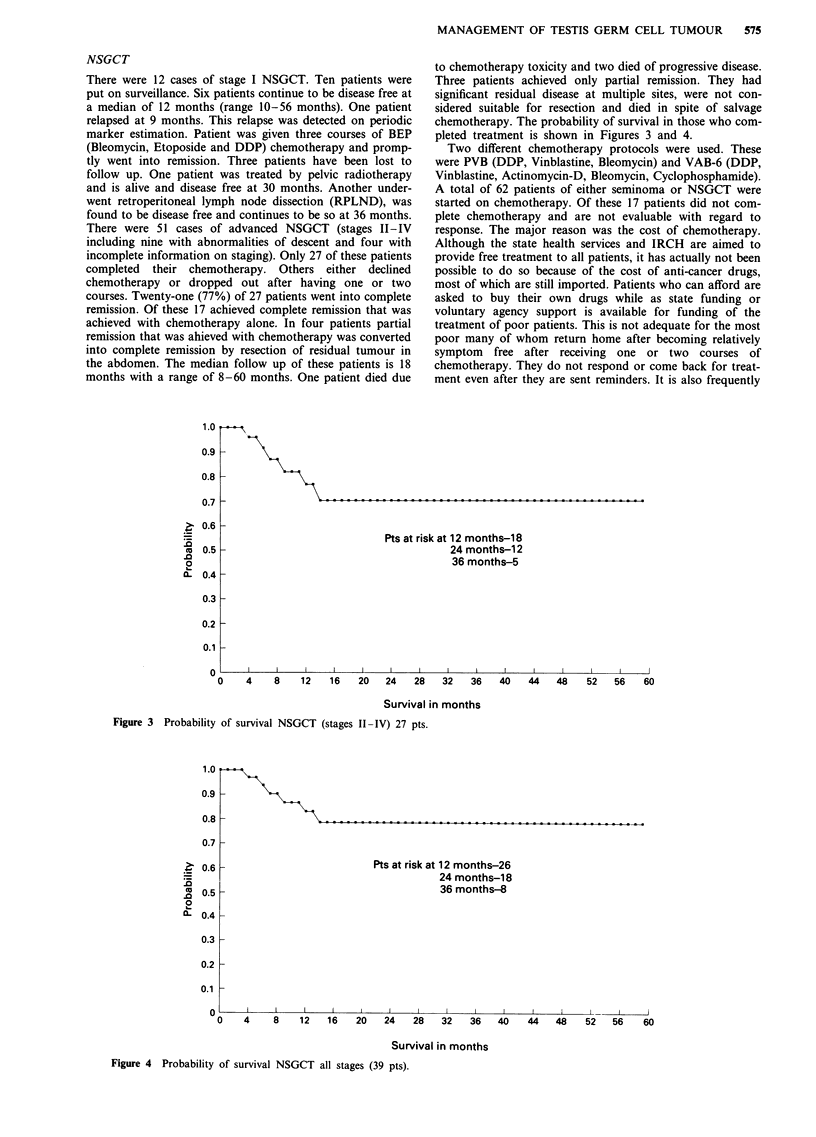

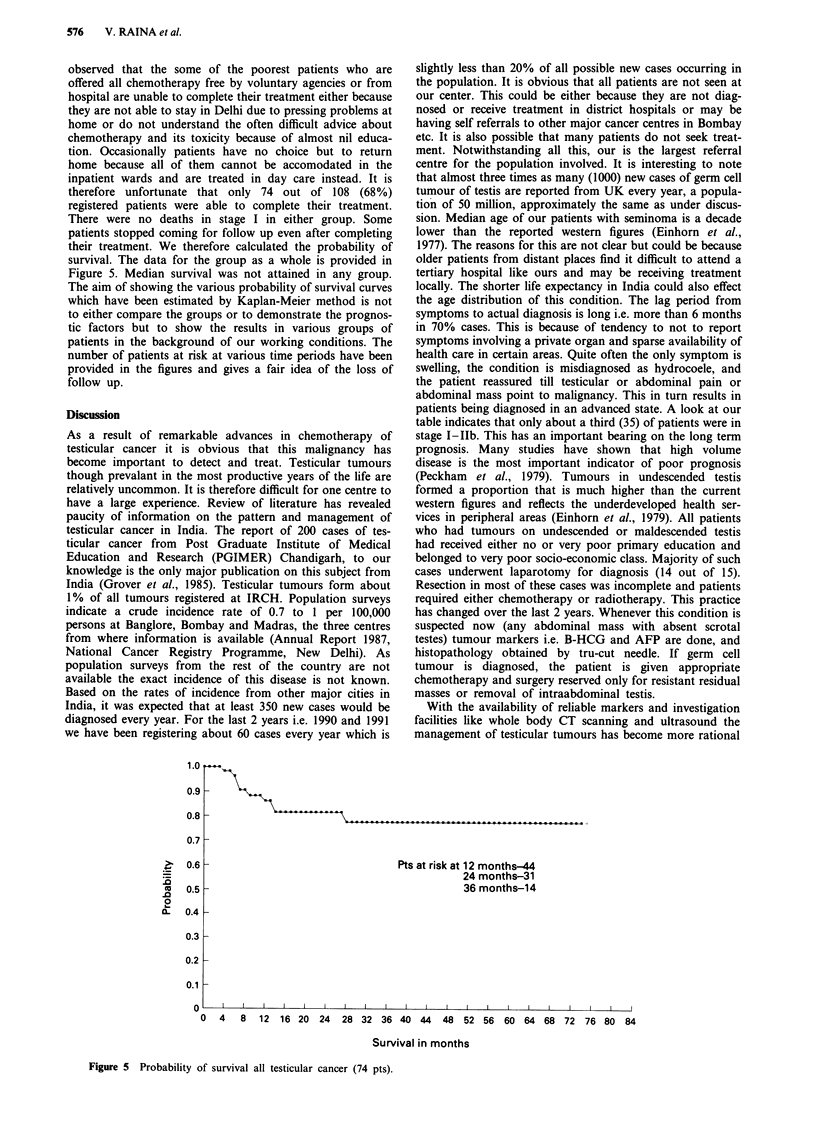

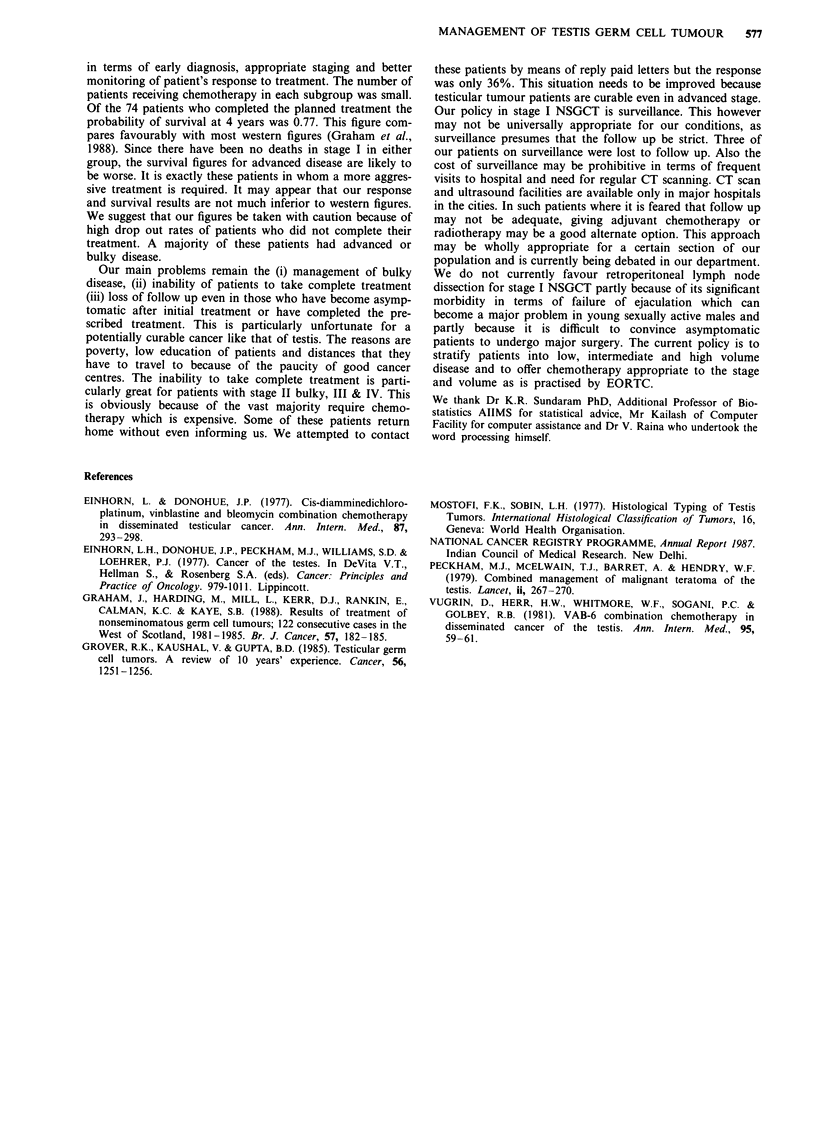


## References

[OCR_00645] Einhorn L. H., Donohue J. (1977). Cis-diamminedichloroplatinum, vinblastine, and bleomycin combination chemotherapy in disseminated testicular cancer.. Ann Intern Med.

[OCR_00657] Graham J., Harding M., Mill L., Kerr D. J., Rankin E., Calman K. C., Kaye S. B. (1988). Results of treatment of non seminomatous germ cell tumours; 122 consecutive cases in the West of Scotland, 1981-1985.. Br J Cancer.

[OCR_00663] Grover R. K., Kaushal V., Gupta B. D. (1985). Testicular germ cell tumors. A review of 10 years' experience.. Cancer.

[OCR_00677] Peckham M. J., McElwain T. J., Barrett A., Hendry W. F. (1979). Combined management of malignant teratoma of the testis.. Lancet.

[OCR_00682] Vugrin D., Herr H. W., Whitmore W. F., Sogani P. C., Golbey R. B. (1981). VAB-6 combination chemotherapy in disseminated cancer of the testis.. Ann Intern Med.

